# Chicken Essence Improves Exercise Performance and Ameliorates Physical Fatigue

**DOI:** 10.3390/nu6072681

**Published:** 2014-07-18

**Authors:** Wen-Ching Huang, Ching-I Lin, Chien-Chao Chiu, Yi-Ting Lin, Wei-Kai Huang, Hui-Yu Huang, Chi-Chang Huang

**Affiliations:** 1 Graduate Institute of Athletics and Coaching Science, National Taiwan Sport University, Taoyuan 33301, Taiwan; E-Mail: magicpica521@gmail.com; 2 Department of Nutrition and Health Sciences, Kainan University, Taoyuan 33857, Taiwan; E-Mail: cilin@mail.knu.edu.tw; 3 Graduate Institute of Sports Science, National Taiwan Sport University, Taoyuan 33301, Taiwan; E-Mail: chiu2295@yahoo.com.tw; 4 Department of Food Science, Nutrition, and Nutraceutical Biotechnology, Shih Chien University, Taipei 10462, Taiwan; E-Mails: linyiting0617@gmail.com (Y.-T.L.); eric29345876@gmail.com (W.-K.H.)

**Keywords:** ergogenic aid, supplement, glycogen, grip strength, lactate, ammonia

## Abstract

Chicken essence (CE) is a liquid nutritional supplement made from cooking whole chickens. In traditional Chinese medicine, CE is used to support health, promote healing, increase metabolism, and relieve fatigue. However, few studies have examined the effect of CE on exercise performance and physical fatigue. We aimed to evaluate the potential beneficial effects of CE on fatigue and ergogenic functions following physical challenge in mice. Male ICR mice were divided into four groups to receive vehicle or CE by oral gavage at 0, 845, 1690, or 4225 mg/kg/day for 4 weeks. Exercise performance and anti-fatigue function were evaluated by forelimb grip strength, exhaustive swimming time, and levels of physical fatigue-related biomarkers serum lactate, ammonia, glucose, and creatine kinase (CK) after physical challenge. CE supplementation dose-dependently elevated endurance and grip strength. CE supplementation significantly decreased lactate, ammonia, and CK levels after physical challenge. Tissue glycogen content, an important energy source for exercise, was significantly increased with CE supplementation. In addition, CE supplementation had few subchronic toxic effects. The supplementation with CE can have a wide spectrum of bioactivities on health promotion, performance improvement and anti-fatigue.

## 1. Introduction

In traditional Chinese medicine, chicken essence (CE) is a liquid nutritional supplement made from cooking whole chickens. It is used for strengthening the bones and muscles, invigorating the spleen and stomach, and enhancing vigor. As compared with other kinds of meat, chicken contains higher protein and is richer in trace elements, amino acids, carnosine, and creatinine, and these ingredients can be extracted into broth or soup. CE differs in ingredient proportions and bioactivities from chicken broth because the processing involves high-temperature extraction, centrifugation, vacuum concentration, and sterilization. CE contains some special nutrients such as carnosine and anserine, taurine, vitamins, minerals, trace elements, and indispensable amino acids [1].

In previous reports related to physiological functions, CE exhibited bioactivities of anti-stress or anti-mental fatigue via cortisol regulation [2] and activation of the central histaminergic system [3]. As well, restraint stress significantly decreased blood insulin, glycogen synthesis, and lipoprotein lipase activity in mice. CE supplementation increased insulin level, lipase activity, and glycogen synthesis in stressed mice [4], which suggests its benefits for stress-induced fatigue by preventing stress-mediated dysfunction in lipid and glucose metabolism. CE supplementation had other beneficial effects on immune function [5], lactation [6], hypertension [7], anemia [8], buffering action [9], circadian clocks resetting process [10] and chemotherapy-induced hematopoietic suppression [11]. However, the mechanisms underlying the bioactivities of CE could be complicated and may be regulated by combined actions of many active components [12].

Fatigue, characterized as physical and/or mental tiredness, could have deleterious effects on work efficiency/performance, physical activities, life quality, and social relationships. Fatigue can be further categorized as secondary, physiologic, or chronic. Secondary fatigue is caused by lack of sleep, low mood, stress, nutritional imbalance, insufficient exercise, or side effects of medication. Physiological fatigue is caused by inadequate rest, physical effort or mental strain and could be classified as peripheral and central fatigue [13]. For chronic fatigue, the etiology is unclear and the syndrome involves a persistent unexplainable fatigue lasting for more than 6 months [14]. Physiological fatigue can be generally alleviated with sufficient rest. CE-related studies of physiological fatigue have mainly related to central or mental fatigue via mediation of histamine, 5-HT (serotonin), 5-hydroxyindoleacetic acid (5-HIAA) or other neurotransmitter pathways [15].

Few reports have addressed the effect of CE supplementation on exercise performance and physical fatigue. Previous study found that CE supplementation could benefit levels of lactate and ammonia metabolites in the recovery phase after one-time exhaustive exercise [16]. We aimed to evaluate the potential ergogenic and anti-fatigue effects of 28 days CE supplementation in mice and examined the subchronic toxic effects of the optimal dose of CE for health promotion.

## 2. Results

### 2.1. Effect of CE Supplementation on Forelimb Grip Strength

**Figure 1:**
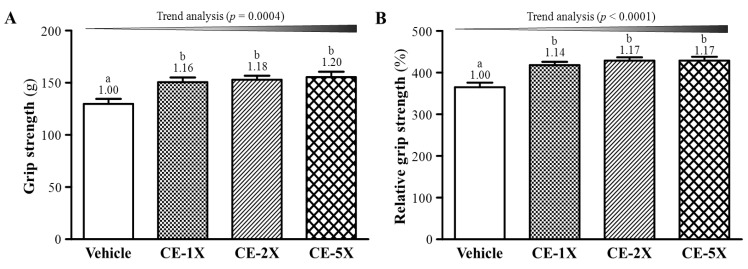
Effect of 4-week chicken essence (CE) supplementation on absolute forelimb grip strength (**A**) and forelimb grip strength (%) relative to body weight (**B**). Data are mean ± SEM for *n* = 10 mice per group. Bars with different letters (a, b) are significantly different at *p* < 0.05. Numbers above the bars are the fold increase from vehicle. Vehicle (water); 845 mg/kg CE (CE-1X); 1690 mg/kg CE (CE-2X); 4225 mg/kg CE (CE-5X).

Absolute grip strength was lower for vehicle- than CE-treated groups (all *p* < 0.005; Figure 1A). Previous study has shown a positive correlation between grip strength and anthropometric factors such as age, weight, body mass index and waist circumference [17]. Therefore, we divided grip strength by BW for relative grip strength and still found lower grip strength for vehicle- than CE-treated groups (Figure 1B). For the trend analysis, absolute and relative grip strength dose-dependently increased with CE supplementation (*p* = 0.0004 and *p* < 0.0001, respectively).

### 2.2. Effect of CE Supplementation on Exercise Performance

**Figure 2:**
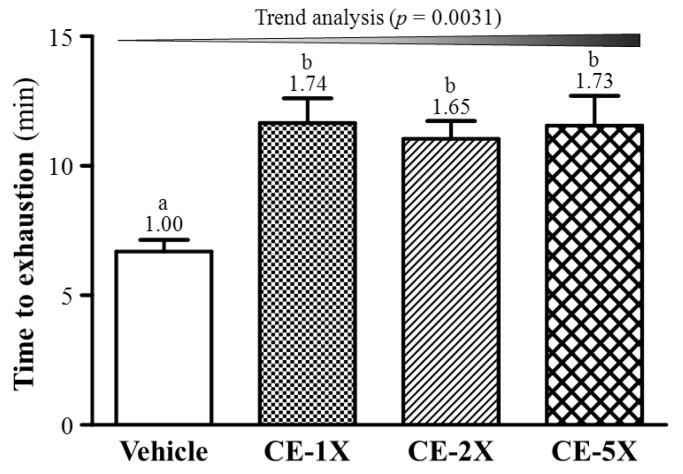
Effect of 4-week CE supplementation on exhaustive swimming time. Data are mean ± SEM for *n* = 10 mice per group. Bars with different letters (a, b) are significantly different at *p* < 0.05.

Endurance with an exhaustive swimming test was higher in mice treated with CE supplementation than vehicle (Figure 2). For the trend analysis, endurance swimming time dose-dependently increased with the CE supplementation (*p* = 0.0031) and swimming time was longer with CE than vehicle supplementation by about 1.65-(*p* = 0.0009) to 1.74-fold (*p* = 0.0002).

### 2.3. Effect of CE Supplementation on Fatigue-Related Biochemical Variables after Acute Exercise Challenge

**Figure 3:**
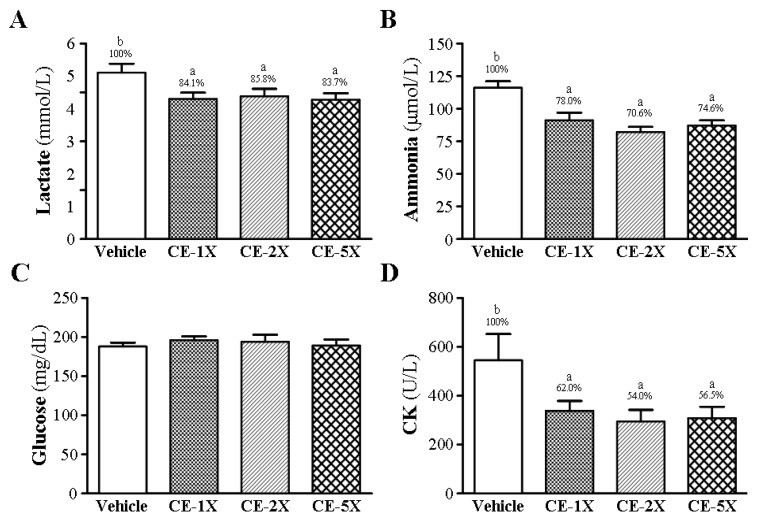
Effect of 4-week CE supplementation on serum levels of lactate (**A**); ammonia (**B**); glucose (**C**) and creatine kinase (CK) (**D**) after a 15-min swimming exercise challenge. Data are mean ± SEM for *n* = 10 mice per group. Bars with different letters (a, b) are significantly different at *p* < 0.05. Percentages above bars show percentage of vehicle.

Lactate levels were higher with vehicle than CE supplementation by about 16.1% (*p* < 0.05) after acute exercise challenge (Figure 3A) and serum ammonia levels were higher (Figure 3B). Values for the CE-1X, CE-2X, and CE-5X groups were significantly lower, by 22% (*p* = 0.001), 29.4% (*p* < 0.0001), and 25.4% (*p* = 0.0002), respectively, than that with vehicle, and ammonia level dose-dependently decreased with CE supplementation with significant trend analysis (*p* < 0.0001).

Levels of serum glucose did not differ between treatment groups. However, blood glucose level was higher but not significantly (*p* > 0.05) (Figure 3C). CK levels were higher with vehicle than CE supplementation (Figure 3D).

### 2.4. Effect of CE Supplementation on Tissue Glycogen Level

**Figure 4:**
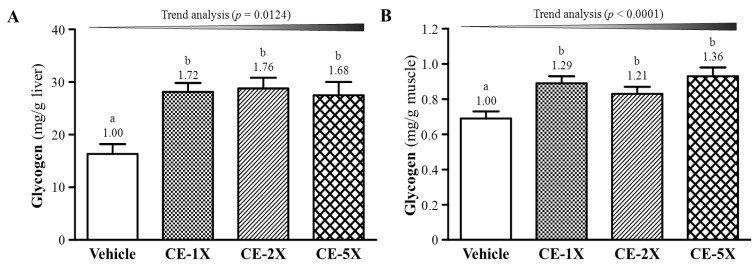
Effect of 4-week CE supplementation on hepatic (**A**) and muscle (**B**) glycogen level. Data are mean ± SEM for *n* = 10 mice per group. Bars with different letters (a, b) are significantly different at *p* < 0.05.

Hepatic glycogen levels were greater with CE than vehicle supplementation (Figure 4A) and muscle glycogen content (Figure 4B) was greater. CE supplementation dose-dependently increased liver and muscle glycogen content on trend analysis (*p* = 0.0124). On trend analysis, CE supplementation dose-dependently increased muscle glycogen levels (*p* < 0.0001).

### 2.5. Subchronic Toxicity Evaluation of CE Supplementation

**Figure 5:**
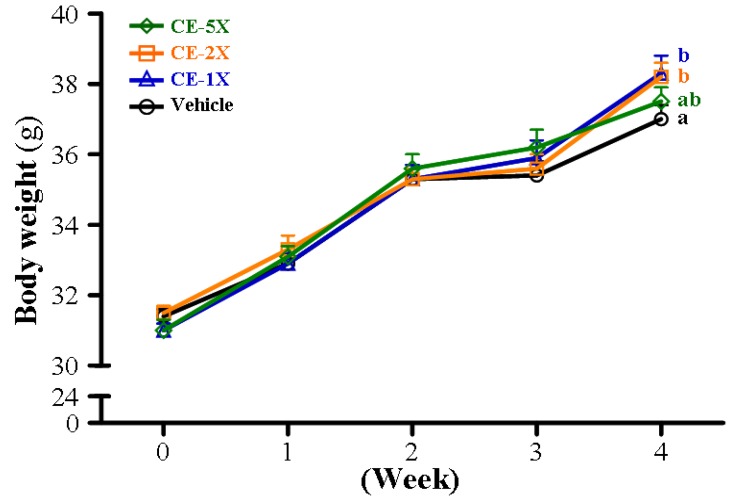
Effect of 4-week CE supplementation on body weight. Data are mean ± SEM for *n* = 10 mice per group. Different letters (a, b) indicate significant difference at *p* < 0.05.

Subchronic toxic effects of CE supplementation were evaluated by behavior, diet, growth curve, organ weight, biochemical assessments and histopathology. The vehicle and CE supplementation groups did not differ in behavior during treatment. The final BW was higher with CE-1X (*p* = 0.039) and CE-2X (*p* = 0.049) than vehicle treatment (Figure 5 and Table 1). The daily intake of diet and water was significantly increased by 1.04-fold (*p* = 0.0022) and 1.06- (*p* = 0.0132), respectively, with CE-2X than vehicle treatment. The weight of organs including live, muscle, kidney, testis, epididymal fat pad (EFP), and brown adipose tissue (BAT) did not differ among groups (Table 1). Levels of biochemical factors, including AST, ALT, ALP, LDH, albumin, TP, TC, TG, glucose, and CK, did not differ among groups (*p* > 0.05, Table 2). However, levels of UA, BUN, and creatinine differed between CE and vehicle supplementation (Table 2). UA levels dose-dependently decreased with CE supplementation, with significance on trend analysis (*p* < 0.0001). The BUN level was greater with CE-2X and CE-5X than vehicle supplementation. The creatinine level was greater with all CE doses than vehicle supplementation. Both BUN and creatinine levels were dose-dependently increased with CE supplementation, with significance on trend analysis (*p* < 0.0001).

**Figure 6:**
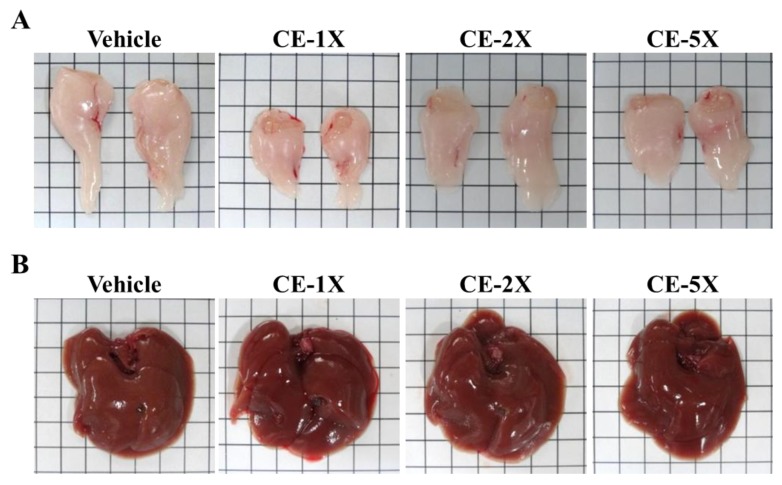
Effect of CE supplementation on epididymal fat pad (EFP; ventral view) (**A**) and liver (**B**) in mice seen by photographs.

**Figure 7:**
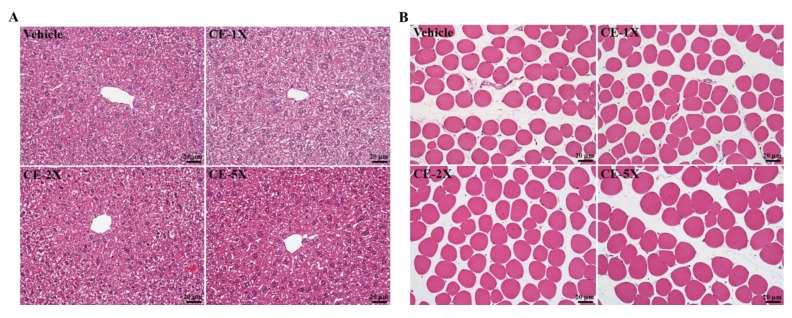
Effect of CE supplementation on histomorphologic features of the liver (**A**) and soleus muscle (**B**) in mice. Specimens were photographed under a light microscope. (H&E stain, magnification: 200×; bar, 20 μm).

Liver and muscle showed no lesions or pathological changes attributable to CE treatment (Figure 6 and Figure 7). In addition, the respective data for soleus muscle mean fiber cross-sectional area (CSA) were 410 ± 22, 402 ± 16, 405 ± 24, and 399 ± 23 μm^2^ in the vehicle, CE-1X, CE-2X, and CE-5X groups, and there were no significant changes in the CSA among each group. We also found no gross abnormalities attributed to CE treatment when weighing organs. Our subchronic toxicity assays revealed that the optimized and reasonable doses of CE we used may have relevant physiological benefits for health promotion.

**Table 1:** General characteristics of mice treated with chicken essence (CE) or vehicle at the end of the experiment.

Characteristics	Vehicle	CE-1X	CE-2X	CE-5X	Trend Analysis *p* Value
Food intake (g/mouse/day)	6.3 ± 0.1 ^b^	6.4 ± 0.0 ^bc^	6.5 ± 0.1 ^c^	5.9 ± 0.1 ^a^	0.0256 (↓)
Water intake (mL/mouse/day)	7.7 ± 0.2 ^a^	8.1 ± 0.2 ^ab^	8.2 ± 0.2 ^b^	8.2 ± 0.2 ^b^	0.0133 (↑)
Initial BW (g)	31.4 ± 0.2	31.0 ± 0.2	31.5 ± 0.2	31.0 ± 0.3	0.6678
Final BW (g)	37.0 ± 0.4 ^a^	38.3 ± 0.5 ^b^	38.2 ± 0.4 ^b^	37.5 ± 0.4 ^ab^	0.3178
Liver (g)	2.16 ± 0.05	2.17 ± 0.05	2.17 ± 0.04	2.11 ± 0.05	0.7186
Muscle (g)	0.38 ± 0.01	0.39 ± 0.01	0.40 ± 0.01	0.39 ± 0.01	0.2168
Kidney (g)	0.68 ± 0.01 ^ab^	0.67 ± 0.01 ^a^	0.69 ± 0.01 ^ab^	0.72 ± 0.02 ^b^	0.0603
Testis (g)	0.25 ± 0.01	0.24 ± 0.01	0.24 ± 0.01	0.24 ± 0.00	0.2719
EFP (g)	0.54 ± 0.03	0.54 ± 0.02	0.52 ± 0.04	0.60 ± 0.03	0.3662
BAT (g)	0.126 ± 0.007	0.124 ± 0.004	0.131 ± 0.005	0.120 ± 0.004	0.5540

Data are mean ± SEM for *n* = 10 mice per group. Data in the same row with different superscript letters (^a^, ^b^ and ^c^) differ significantly, *p* < 0.05 by one-way ANOVA. Muscle mass includes both gastrocnemius and soleus muscles in the back part of the lower legs. BW: body weight. EFP: epididymal fat pad. BAT: brown adipose tissue (CE-1X: 845 mg/kg CE. CE-2X: 1690 mg/kg CE. CE-5X: 4225 mg/kg CE).

**Table 2:** Biochemical analysis of vehicle and CE treatment groups at the end of the experiment.

Parameters	Vehicle	CE-1X	CE-2X	CE-5X	Trend Analysis *p* Value
AST (U/L)	84 ± 12	78 ± 4	76 ± 3	79 ± 5	0.3850
ALT (U/L)	67 ± 10 ^b^	47 ± 3 ^a^	56 ± 4 ^ab^	54 ± 6 ^ab^	0.8386
ALP (U/L)	82 ± 5	76 ± 6	73 ± 5	78 ± 4	0.5927
LDH (U/L)	383 ± 58	336 ± 33	375 ± 29	361 ± 37	0.5300
Albumin (g/dL)	3.6 ± 0.1	3.7 ± 0.1	3.6 ± 0.1	3.7 ± 0.1	0.2990
TP (g/dL)	4.9 ± 0.0	5.0 ± 0.1	5.0 ± 0.0	5.0 ± 0.1	0.4529
BUN (mg/dL)	20.8 ± 0.9 ^a^	20.2 ± 0.3 ^a^	23.3 ± 0.8 ^b^	25.5 ± 0.4 ^c^	<0.0001 (↑)
Creatinine (mg/dL)	0.16 ± 0.01 ^a^	0.28 ± 0.01 ^b^	0.35 ± 0.02 ^c^	0.51 ± 0.03 ^d^	<0.0001 (↑)
UA (mg/dL)	1.9 ± 0.1 ^b^	1.4 ± 0.1 ^a^	1.5 ± 0.1 ^a^	1.3 ± 0.1 ^a^	<0.0001 (↓)
TC (mg/dL)	109 ± 5	105 ± 4	117 ± 6	108 ± 5	0.2260
TG (mg/dL)	110 ±6	118 ± 9	119 ± 8	112 ± 5	0.7562
Glucose (mg/dL)	175 ± 5	187 ± 6	185 ± 5	179 ± 3	0.6398
CK (U/L)	369 ± 171	363 ± 96	302 ± 65	376 ± 56	0.0989

Data are mean ± SEM for *n* = 10 mice per group. Data in the same row with different superscript letters (^a^, ^b^ and ^c^) differ significantly, *p* < 0.05 by one-way ANOVA. AST, aspartate aminotransferase; ALT, alanine aminotransferase; ALP, alkaline phosphatase; CK, creatine kinase; LDH, lactate dehydrogenase; TP, total protein; BUN, blood urea nitrogen; UA, uric acid; TC, total cholesterol; TG, triacylglycerol.

## 3. Discussion

In previous studies, daily CE supplementation could be effective for recovery from or performance in mental fatigue tasks, including 2-back test trials or mental arithmetic and short-term memory tests [2,18]. Few studies have addressed physical endurance with the supplementation. We evaluated physical activities after CE supplementation in mice by exhaustive swimming and grip strength. The exhaustive swimming test was performed by equivalent percentage loading of BW along with forced swimming until exhaustion and the grip strength represented the power output of muscle strength for the maximum values. This method is widely used to evaluate endurance capacity for exercise performance [19,20]. As shown in Figure 1 and Figure 2, CE supplementation could significantly increase endurance with this swimming aerobic exercise and elevated grip strength without training intervention.

Muscle strength can be improved by resistance training via muscular and neuromuscular adaption [21,22], and nutritional supplements could also play an important role on amelioration of muscle damage caused by oxidative stress or myogenesis assistance for muscle adaption [23,24]. Therefore, regulatory training may efficiently elevate muscle strength or adaption combined with CE supplementation. Distinguishing which components of CE have beneficial effects on physical fatigue is difficult because CE is composed of many different active substances. The CE, containing bioactivity compounds such as imidazole dipeptides, may postpone the physical-induced fatigue via antioxidant activity [25]. However, the benefits of CE in different types of exercise need further investigation.

Exercise-induced physical fatigue could be evaluated by assessing serum biochemicals, including lactate, ammonia, creatine kinase, and glucose, as we previously reported [19,20,26]. Lactate is a well-known glucose metabolite in anaerobic glycolysis for energy needs of muscle tissue during high intensive exercise. Lactate will be accumulated with extended duration of exercise and pH value will decrease, which can result in various metabolic and physiological side effects on glycolytic processes and calcium ion release related to muscle contractions [27]. Another important metabolite, ammonia, will significantly increase with intensity or prolonged time during exercise and the exercise-induced hyperammonaemia can be reduced by nutritional supplementation [28]. During energy metabolism for exercise, ammonia is generated by different sources. The immediate source of ammonia production is the purine nucleotide cycle [29] and ammonia is substantially elevated during intensive or prolonged exercise when the rate of ATP utilization may exceed the rate of ATP production. The ammonia toxicity may affect continuing coordinated activity in critical regions of the central nervous system. In our Figure 3A,B, the levels of lactate and ammonia significantly decreased with CE supplementation after immediate acute exercise in mice.

Glucose levels are increased by the combined actions of epinephrine, norepinephrine, glucagon and cortisol during exercise. The physiological function of insulin helps cells uptake glucose via activation of PI-3 kinase to increase the translocation of GLUT4, the glucose transporter, but the insulin concentration declines during prolonged exercise. Therefore, the activation of glucose uptake differs between insulin and exercise-induced mechanisms or pathways [30,31]. During exercise, the glycolysis form tissue glycogen is the major energy substance and uptake is elevated by 26% due to muscle contractions [32]. Blood glucose levels are an important index for performance maintenance during exercise. In our Figure 3C, glucose level with CE supplementation did not significantly increase as compared to vehicle supplementation. CE supplementation may upregulate the translocation of GLUT4 protein to efficiently uptake glucose for exercise energy, rather than the glycolysis activation mechanism; however, this needs further verification.

In the phosphagen system, CK catalyzes creatine to convert phosphocreatine (PCr) by ATP consumption. PCr could serve as an energy reservoir for the rapid buffering and regeneration of ATP for short-term energy use. However, intensive or exhaustive exercise will induce oxidative stress, such as production of reactive oxygen species and free radicals, to injure cells or tissues [33,34]. Such injury includes lipid peroxidation, which destroys membrane permeability, cell organization, DNA integrity and function. As well, important enzymes such as LDH, CK, myoglobin, AST, and ALT are released into serum and are considered biomarkers of tissue injury under high-intensity exercise challenge [35]. In previous study, CE supplementation had benefits for lactate and ammonia levels in the recovery phase after exhaustive exercise [16]. Our CE supplementation significantly decreased lactate, ammonia and CK levels after immediate, acute exercise challenge. Therefore, CE could improve the fatigue-related biomarker levels with continuous supplementation.

Exercise performance is determined by energy storage and supply. Glucose, a predominant source of glycolysis for aerobic or anaerobic ATP production, is the major energy source for exercise and is stored as liver and muscle glycogen which could play an important role in maintaining blood-glucose homeostasis [36]. The muscle content of glycogen is a limiting factor for prolonged exercise and nutritional interventions could be beneficial for increasing or maintaining liver or muscle glycogen content before or during exercise [19,37]. Therefore, exercise ability could be directly affected by glycogen storage [38]. Our results in Figure 4 showed a significant increase in tissue glycogen storage with CE supplementation, which could enhance endurance performance.

Chicken broth or soup supplementation has been used for more than 100 years and it is familiar and popular in Southeast China. However, chicken broth is produced by industrial food and processing technology with high quality control as CE for accommodating modern life. Therefore, CE is considered a nutritional supplement, mainly because of its protein, peptides, minerals, multiple amino acids, and trace elements [1] and is widely accepted. Researchers have revealed the many bioactivities of CE, and the mechanisms involved in these bioactivities are complicated. It may be regulated by the combination of many active compounds, not only the components alone [12]. However, limited toxicological reference is available regarding its safety. The no-observed-adverse-effect level of CE could provide optimized dosages for its physiological benefits without health risk for pursuing health preservation. Our results of subchronic toxicity with CE supplementation showed no deleterious effects on growth, diet, survival rate, organ weight, most biochemical assessments, and pathological evaluation in mice as compared with the vehicle. BUN and creatinine levels were higher and UA level was lower with CE than vehicle treatment. According to our previous studies [20,26], serum UA and BUN levels with CE treatment were still within the normal range, even though the data showed that the CE supplementations dose-dependently changed these two indices. However, with CE supplementation, especially CE-5X, the creatinine level was 3.19 times higher than with vehicle treatment. This could be a potential risk for rental disease patients who want obtain physiological benefits with high dosage and long-term CE supplementation.

## 4. Experimental

### 4.1. Materials, Animals, and Experiment Design

CE extract used for supplementation was provided by Jicond Foods (Taichung, Taiwan). CE extract was prepared by good manufacturing practices and hazard analysis and critical-control-point qualified manufacturing. An amount of 6.4 kg black feather native chicken of Taiwan was cooked and extracted by use of water to obtain the 720 g CE extracts. To ensure precise and accurate dosing of animals, heat-sterilized CE extract was lyophilized by freeze-drying to obtain powder extract. The crude extracted powder from CE was stored at −80 °C until used for the experiment. The nutrition facts and total branched-chain amino acids of CE were analyzed by SGS Taiwan, Ltd. and are shown in Table 3. The hydrolyzed amino acid profile was determined by Food Industry Research and Development Institute, Taiwan.

In this study, the dose of CE designed for humans was 4.122 g per day (lyophilized powder), which would be equivalent to a daily recommended dose of CE at 60 mL/serving/day. The mouse CE dose (845 mg/kg) we used was converted from a human equivalent dose (HED) based on body surface area by the following formula from the US Food and Drug Administration: assuming a human weight of 60 kg, the HED for 4.122 (g)/60 (kg) = 0.167 × 12.3 = a mouse dose of 845 mg/kg; the conversion coefficient 12.3 was used to account for differences in body surface area between mice and humans as we described previously [39].

We used male ICR mice (6 weeks old) from BioLASCO Taiwan (Yi-Lan Breeding Center, Yi-Lan County, Taiwan) accredited by the Association for Assessment and Accreditation of Laboratory Animal Care International. Mice were acclimatized and allowed food *ad libitum* for 2 weeks prior to experiments. All animals were given a standard laboratory diet (No. 5001; PMI Nutrition International, Brentwood, MO, USA) and distilled water *ad libitum*, and maintained at 12-h light/12-h dark cycle at room temperature (22 ± 2 °C) and 50%–60% humidity. The bedding was changed and cleaned twice per week. The Institutional Animal Care and Use Committee (IACUC) of National Taiwan Sport University approved all animal experiments in this study, and the study conformed to the guidelines of protocol IACUC-10213 approved by the IACUC ethics committee.

**Table 3:** Nutrition facts, hydrolyzed amino acid profiles and total branched-chain amino acids of chicken essence (CE).

Nutrition Facts	Content
Nutrition Facts	/60 mL CE/Serving
Protein	3.9 g
Fat	0
Saturated fat	0
Trans fat	0
Carbohydrate	0
Sodium	0.0364 g
Total calories	15.6 Kcal
Hydrolyzed amino acid profiles	mg/100 g
Aspartic Acid	322.9
Threonine	127.4
Serine	164.9
Glutamic acid	654.0
Glycine	1105.0
Alanine	464.8
Cystine	8.6
Valine	123.9
Methionine	49.4
Isoleucine	79.1
Leucine	180.2
Tyrosine	183.4
Phenylalanine	97.5
Lysine	248.9
Histidine	142.0
Arginine	395.6
Proline	613.7
Total BCAA	mg/60 g CE
Valine, leucine and isoleucine	443.0

Nutrition Facts and total branched-chain amino acids were analyzed by SGS Taiwan Ltd. The hydrolyzed amino acid profiles were determined by Food Industry Research and Development Institute, Taiwan.

All animals were randomly assigned to four groups (10 mice/group) for oral gavage treatment for 4 weeks: (1) vehicle (water); (2) 845 mg/kg CE (CE-1X); (3) 1690 mg/kg CE (CE-2X); and (4) 4225 mg/kg CE (CE-5X). The vehicle group received the same volume of solution equivalent to body weight (BW). The food intake and water consumption were monitored daily, and BW was recorded weekly.

### 4.2. Swimming Exercise Performance Test

The swim-to-exhaustion exercise test involved constant loads corresponding to 5% of BW to analyze endurance time as we previously described [20,26]. The swimming endurance time was recorded from the beginning to exhaustion, determined by observing loss of coordinated movements and failure to return to the surface within 7 s.

### 4.3. Forelimb Grip Strength

We used a low-force testing system (Model-RX-5, Aikoh Engineering, Nagoya, Japan) to measure forelimb grip strength as we previously described [19,20,26].

### 4.4. Determination of Fatigue-Associated Biochemical Variables

The effect of CE supplementation on fatigue-associated biochemical indices were evaluated post-exercise as we previously described [20,26]. At 1 h after CE supplementation, all animals underwent a 15-min swim test without weight loading. Blood samples were immediately collected and centrifuged at 1500× *g* and 4 °C for 10 min for serum separation. Serum lactate, ammonia, glucose and creatinine kinase (CK) levels were determined by use of an autoanalyzer (Hitachi 7060, Hitachi, Tokyo, Japan).

### 4.5. Clinical Biochemical Profiles

One hour after the last treatment, all mice were killed by 95% CO_2_ asphyxiation, and blood was immediately collected. Serum was separated by centrifugation and clinical biochemical variables, including aspartate aminotransferase (AST), alanine transaminase (ALT), alkaline phosphatase (ALP), lactate dehydrogenase (LDH), CK, albumin, total bilirubin (TBIL), thymidine phosphorylase (TP), blood urea nitrogen (BUN), creatinine, uric acid (UA), total cholesterol (TC), triglycerides (TG) and glucose were measured by use of an autoanalyzer (Hitachi 7060).

### 4.6. Tissue Glycogen Determination and Visceral Organ Weight

The glucose stored form is glycogen that mostly exists in liver and muscle tissue. Liver and muscle tissues were excised after mice were killed and weighed for glycogen content analysis as we described previously [26]. The weights of related visceral organs were recorded.

### 4.7. Histology Staining of Tissues

Tissue samples from each group were photographed with a Cyber-shot (DSC-HX30V, SONY, Tokyo). Liver and muscle tissues (soleus) were minced and fixed in 10% formalin, embedded in paraffin and cut into 4-μm-thick slices for morphology and pathology. Tissue sections were stained with hematoxylin and eosin (H&E) and examined under a light microscope equipped with a CCD camera (BX-51, Olympus, Tokyo, Japan) by a veterinary pathologist.

### 4.8. Statistical Analysis

Data are expressed as mean ± SEM. Statistical differences among groups were analyzed by a one-way analysis of variance (ANOVA) and the Cochran-Armitage test for dose-effect trend analysis with use of SAS v9.0 (SAS Inst., Cary, NC, USA). *p* < 0.05 was considered statistically significance.

## 5. Conclusions

Taken together, we provide evidence-base results here to show that CE could improve physical-induced fatigue and elevate exercise performance in mice, as shown in the Supplementary material. CE could ameliorate exercise-related increases in levels of biomarkers such as lactate and ammonia. It could decrease CK level, a muscle injury biomarker. It could elevate exercise performance by increasing tissue glycogen content. As well, CE could be supplemented with optimized and reasonable doses for physiological benefits. Excess doses could cause potential risk of renal loading with 28 days supplementation. 
